# A Disintegrin and Metalloproteinase 9 Is Involved in Ectodomain Shedding of Receptor-Binding Cancer Antigen Expressed on SiSo Cells 

**DOI:** 10.1155/2014/482396

**Published:** 2014-08-07

**Authors:** Kenzo Sonoda, Kiyoko Kato

**Affiliations:** Department of Obstetrics and Gynecology, Graduate School of Medical Sciences, Kyushu University, Maidashi 3-1-1, Higashi-ku, Fukuoka 812-8582, Japan

## Abstract

In several human malignancies, the expression of receptor-binding cancer antigen expressed on SiSo cells (RCAS1) is associated with aggressive characteristics and poor overall survival. RCAS1 alters the tumor microenvironment by inducing peripheral lymphocyte apoptosis and angiogenesis, while reducing the vimentin-positive cell population. Although proteolytic processing, referred to as “ectodomain shedding,” is pivotal for induction of apoptosis by RCAS1, the proteases involved in RCAS1-dependent shedding remain unclear. Here we investigated proteases involved in RCAS1 shedding and the association between tumor protease expression and serum RCAS1 concentration in uterine cancer patients. A disintegrin and metalloproteinase (ADAM) 9 was shown to be involved in the ectodomain shedding of RCAS1. Given the significant correlation between tumor ADAM9 expression and serum RCAS1 concentration in both cervical and endometrial cancer as well as the role for ADAM9 in RCAS1 shedding, further exploration of the regulatory mechanisms by which ADAM9 converts membrane-anchored RCAS1 into its soluble form should aid the development of novel RCAS1-targeting therapeutic strategies to treat human malignancies.

## 1. Introduction

To date, over 150 scientific reports have been published that concern the biological functions and clinical significance of RCAS1. RCAS1 is a 639 amino acid, type II membrane protein with an N-terminal transmembrane segment and a C-terminal coiled-coil structure that is involved in oligomer formation [1]. Since RCAS1 promotes tumor cell evasion of immune surveillance by inducing apoptosis in immune cells, including peripheral lymphocytes, and also remodels the cancer stromal microenvironment, RCAS1 is believed to contribute to tumor progression [2]. Clinically, RCAS1 expression is significantly higher in cancerous tissues relative to normal tissues [3], and its expression increases during the progression from precancerous lesions to cancer [4, 5]. RCAS1 expression is associated with several clinicopathological parameters of human malignancies, including histological type, differentiation, tumor size, stage, depth of invasion, lymphovascular space involvement, lymph node metastasis, and positive peritoneal cytological results [6]. In addition, RCAS1 is a negative predictor of overall survival in 15 different kinds of cancers occurring in the brain, oral cavity, lung, pleural mesothelium, esophagus, stomach, bile duct, gallbladder, pancreas, colon, gastrointestinal mesenchyme, kidney, prostate, uterine cervix, and endometrium [2].

RCAS1 is shed in the serum and pleural effusion and as such may be a useful biomarker for human cancer due to its ability to predict the results of medical treatments [7, 8]. During the conversion from a membrane-anchored to a shedded protein, RCAS1 undergoes proteolytic processing known as “ectodomain shedding” [9]. Ectodomain shedding affects the biological activity of membrane proteins such as growth factors, growth factor receptors, cell-adhesion molecules, and extracellular matrix proteins by altering their localization and mode of action [10]. For membrane-anchored growth factors, ectodomain shedding can convert them into diffusible factors and greatly influence their functions. The membrane-anchored form of Spitz, a transforming growth factor (TGF)-*α*-like molecule that is an epidermal growth factor receptor (EGFR) ligand in* Drosophila*, is inactive but is activated following proteolytic cleavage to yield a soluble protein [11]. In contrast, membrane-anchored c-kit ligand [12] and ephrins [13] are fully functional, while their soluble forms exhibit little or no biological activity. RCAS1 induces apoptosis mainly via its shedded form and not the membrane-anchored form. Therefore, regulation of the conversion of membrane-anchored proteins into their soluble form would be an important way to modify the action of these molecules, including RCAS1. Accumulating evidence demonstrated a role for proteolytic enzymes such as matrix metalloproteinase (MMP), ADAM, and the closely related ADAM with thrombospondin motifs (ADAMTSs) in cancer development and progression [14, 15]. MMPs, ADAMs, and ADAMTSs play a crucial role during all stages of cancer progression, from initiation to metastatic spreading. Besides their role in shedding of plasma membrane-associated proteins and intracellular signaling, these proteases regulate growth factor activation, angiogenesis, inflammation, and apoptosis [16, 17].

Although RCAS1 induces apoptosis mainly after being converted to its shedded form, the proteases involved in this ectodomain shedding remain unclear. To understand more clearly the regulation of membrane-anchored RCAS1 conversion, we sought to (1) identify key proteases involved in RCAS1 shedding and (2) determine whether there is an association between tumor protease expression and serum RCAS1 concentration in cervical and endometrial cancer patients.

## 2. Materials and Methods

### 2.1. Cell Lines

The human uterine cervical adenocarcinoma cell line SiSo, human breast adenocarcinoma cell line MCF-7, human chronic myelogenous leukemia cell line K562, and mouse embryo fibroblast L cells were maintained in RPMI 1640 medium supplemented with 100 units/mL penicillin G, 100 *μ*g/mL streptomycin, and 10% fetal bovine serum (FBS) (ICN Biomedical, Irvine, CA) in a humidified incubator (37°C, 5% CO_2_). Both SiSo and MCF-7 cells express membrane-anchored RCAS1, but RCAS1 shedding is undetectable in MCF-7 culture supernatants [9]. RCAS1 expression and shedding are undetectable in K562 cells and L cells that express a putative RCAS1 receptor [1]. We established the SiSo cell line from uterine cervical adenocarcinoma [18] and the other cell lines were purchased from the American Type Culture Collection.

### 2.2. Patients and Surgical Specimens

Tissue samples from cervical and endometrial cancer patients were used for immunohistochemical analysis. All patients had received medical treatment between April 2010 and January 2013 at the Department of Obstetrics and Gynecology, Kyushu University Hospital (Table 1). The mean patient age was 43 years (range of 24–81 years) for cervical cancer and 58 years (range of 37–84 years) for endometrial cancer. The histologic subtypes were 33 cases of squamous cell carcinoma and 14 cases of adenocarcinoma in cervical cancer and 48 cases of endometrioid adenocarcinoma (24 cases of grade 1; 15 cases of grade 2; 9 cases of grade 3) in endometrial cancer. Cases were classified into stages as follows: 36 cases: stage I; 11 cases: stage II in cervical cancer; 28 cases: stage I; 5 cases: stage II; 13 cases: stage III; 2 cases: stage IV in endometrial cancer. These specimens were graded according to the 2008 International Federation of Gynecology and Obstetrics criteria. All specimens were fixed, embedded in paraffin, and stained with hematoxylin and eosin for determination of histologic subtype. Informed consent was obtained from all patients in this study. This study protocol was approved by the Ethical Committee of Kyushu University.

### 2.3. Evaluation of RCAS1 Expression by Flow Cytometry

To evaluate RCAS1 expression, flow cytometric analysis was performed using the monoclonal antibody 22-1-1 (MBL, Nagoya, Japan) that recognizes human RCAS1. Briefly, cells were harvested followed by incubation with 22-1-1 antibody on ice for 45 minutes. After the cells were washed, they were incubated for 45 minutes with fluorescein isothiocyanate-conjugated goat anti-mouse IgM antibody (Pierce, Rockford, IL) on ice. The cells were again washed, and flow cytometric analysis was performed using FACScan (Becton Dickinson, San Jose, CA).

### 2.4. Enzyme-Linked Immunosorbent Assay (ELISA)

We measured RCAS1 concentrations of the cell culture supernatant in triplicate with an ELISA kit (MBL, Nagoya, Japan), according to the manufacturer's instructions. The RCAS1 ELISA kit was applied in earlier investigations of the clinical significance of RCAS1 in uterine cancer [19]. The sensitivity of the RCAS1 assay was 0.008 U/mL. Mean concentrations of triplicate measurements were calculated.

### 2.5. Generation of SiSo Cells Expressing ADAM9 Small Interfering Ribonucleic Acid (siRNA)

To construct specific siRNA for ADAM9, oligonucleotides were synthesized and purified by Takara Bio (Shiga, Japan) as follows: sense 5′-GGAGAUUUGGACCAAUGGATT-3′ and antisense 5′-UCCAUUGGUCCAAAUCUCCTT-3′. The target specificity of these sequences was confirmed by a BLAST search (http://www.ncbi.nlm.nih.gov/gene/). Homologous siRNA oligonucleotides were dissolved in buffer (100 mM potassium acetate, 30 mM 4-(2-hydroxyethyl)-1-piperazineethanesulfonic acid (HEPES) plus potassium hydroxide, 2 mM magnesium acetate, pH 7.4) to a final concentration of 20 *μ*M, heated to 90°C for 60 seconds, and incubated at 37°C for 60 minutes before use to disrupt higher order aggregates formed during synthesis. The complexes of transfection reagent (Invitrogen, Carlsbad, CA) plus siRNA were added to SiSo culture dishes. Assays were performed 48 hours after treatment. A nontargeting control siRNA that did not have homology with known gene targets in mammalian cells was also used. The control siRNA GC content was 38.1%, which is identical to that of the siRNAs constructed here.

### 2.6. Generation of MCF-7 Cells Stably Expressing ADAM9

MCF-7 cells were transfected with the expression vector pEF-BOS carrying human ADAM9 complementary deoxyribonucleic acid (cDNA) or an empty pEF-BOS vector using Lipofectamine 2000 reagent (Invitrogen). Transfected cells were selected with 250 *μ*g/mL G418. A clone was established after transfection with ADAM9 cDNA that was named MCF-7/ADAM9. One clone was isolated after transfection with the pEF-BOS vector alone.

### 2.7. Western Blot Analysis

Cells were lysed in radioimmunoprecipitation assay buffer (1% Triton-X, 1% sodium deoxycholate, 0.1% sodium dodecyl sulfate (SDS), 150 mM NaCl, 50 mM Tris (pH 8.0), 0.2 unit/mL aprotinin, 2 *μ*g/mL leupeptin, 1 *μ*g/mL pepstatin A, 2 mM phenylmethylsulfonyl fluoride, and 1 mM sodium orthovanadate). Extracts were then subjected to SDS-polyacrylamide gel electrophoresis (PAGE) and immunoblotting analysis after transfer to Immobilon-P transfer membranes (Millipore Corporation, Bedford, MA). Membranes were probed with several antibodies including rabbit anti-ADAM9 (Chemicon, Temecula, CA) and mouse anti-*β*-actin (Novus Biologicals, Littleton, CO) antibodies. Peroxidase-conjugated goat anti-rabbit (Southern Biotech, Birmingham, AL) or anti-mouse IgG (Chemicon) was used as a secondary antibody.

### 2.8. Microarray Analysis

Hybridization targets for the GeneChip Human Gene 1.0 ST array were prepared using the GeneChip WT cDNA Synthesis and Amplification Kit, GeneChip Sample Cleanup Module and GeneChip WT Terminal Labeling Kit according to the manufacturer's protocol (Affymetrix, Santa Clara, CA). Briefly, the cells were harvested in the logarithmic growth phase and total RNA was extracted. Total RNA (100 ng) was converted into double-stranded cDNA (1st-cycle), and the complementary RNA (cRNA) was synthesized by* in vitro* transcription. After purification and measurement of cRNA, 10 *μ*g was converted into single-stranded DNA (ssDNA, 2nd cycle), of which 5.5 *μ*g was fragmented and labeled. The ssDNA was hybridized to the array described above for 16 hours at 45°C. Following hybridization, the array was automatically washed and stained with the GeneChip Hybridization, Wash and Stain Kit. The Probe Array was scanned using the GeneChip Scanner 3000 7G. Microarray analysis was performed three times using three independent cell cultures.

### 2.9. Evaluation of Apoptotic Cell Death

Induction of apoptosis in K562 cells was evaluated by coculturing with four effecter cell types, including SiSo, MCF-7, MCF-7/ADAM9, or L cell. Each effecter cell (1 × 10^5^ cells/well) and K562 target cells were coincubated in a 6-well plate at 1–20 : 1 effecter/target (E/T) ratio. To enhance tight cell-cell contacts, the plates were centrifuged once after coculture initiation. The suspended cells were harvested and stained with the Annexin V-PE apoptosis detection kit (MBL) on days 1–4 after beginning the experiment. Flow cytometric analysis was performed to measure the number of apoptotic cells. To discriminate K562 cells from effecter cells, K562 cells were stained using the green fluorescence cell linker PKH kit (Sigma, St. Louis, MI) before coculture initiation. Evaluation of K562 cell apoptosis was performed three times using three independent cell cultures.

### 2.10. Immunocytochemical Detection of RCAS1 and ADAM9 Association

A proximity ligation assay (PLA) to detect an association between RCAS1 and ADAM9 was carried out using a Duolink detection kit (Olink Bioscience, Uppsala, Sweden). Briefly, SiSo, MCF-7, ADAM9 siRNA-transfected SiSo, and RCAS1 siRNA-transfected SiSo cells [20] were seeded into 8-well chamber slides. On the next day, cultures were fixed in 90% ethanol/5% acetic acid and subjected to PLA. Slides were incubated with mouse anti-RCAS1 (MBL) and rabbit anti-ADAM9 (Chemicon) antibodies and then secondary antibodies conjugated to unique DNA probes (PLA probe MINUS and PLUS) were added. Ligation and circularization of the DNA were followed by a rolling circle amplification step, and reactions were detected by a complementary Tex613 fluorophore-labeled DNA linker [21]. Slides were evaluated using an LSM 510 META confocal microscope (Zeiss, Jena, Germany).

### 2.11. Immunohistochemistry

For immunohistochemical analyses, one or two representative samples selected for each case were analyzed by means of the streptavidin-biotin method. The 22-1-1 antibody (MBL) or rabbit anti-ADAM9 antibody (Abcam, Cambridge, MA) was applied as the primary antibody. Positive control samples were as follows: for RCAS1, cervical adenocarcinoma, which was used to manufacture the 22-1-1 antibody [3], and for ADAM9, breast cancer [22]. We also performed assays without immunized mouse and rabbit immunoglobulins as negative controls. No significant immunohistochemical reaction occurred in the control sections.

Immunohistochemical expression of RCAS1 and ADAM9 was reviewed without knowledge of the clinicopathologic data. Evaluation of expression consisted of an examination of five representative fields, with 1000 tumor cells (200 for each field) being counted via a microscope with a high-power (400x) objective. Tissue sections with more than 5% reactive cells were defined as positive and graded as follows: 1+, 5% to 25% positive cells; 2+, 26% to 50% positive cells; and 3+, 51% to 100% positive cells.

### 2.12. Statistical Analysis

The Fisher's exact (chi-square) test was done to evaluate the association between RCAS1 and ADAM9 expression in tumor tissues resected from uterine cancer patients. The Mann-Whitney test was performed to check differences in antigen expression and secretion between different groups of cells. *P* values of <0.05 were considered statistically significant.

## 3. Results

### 3.1. Differences in Protease Expression between SiSo and MCF-7 Cells

The expression of proteases was compared between SiSo and MCF-7 cells by microarray analysis (Table 2). The ADAM9 expression level was significantly higher in SiSo cells, as shown by relative signals of 1856.6 and 275.1 in SiSo and MCF-7 cells, respectively, which yields a relative ratio of 6.75. No other proteases showing strong expression signals were significantly different between SiSo and MCF-7 cells.

### 3.2. Changes in RCAS1 Expression and Shedding after Gene Transfection

**Figure 1:**
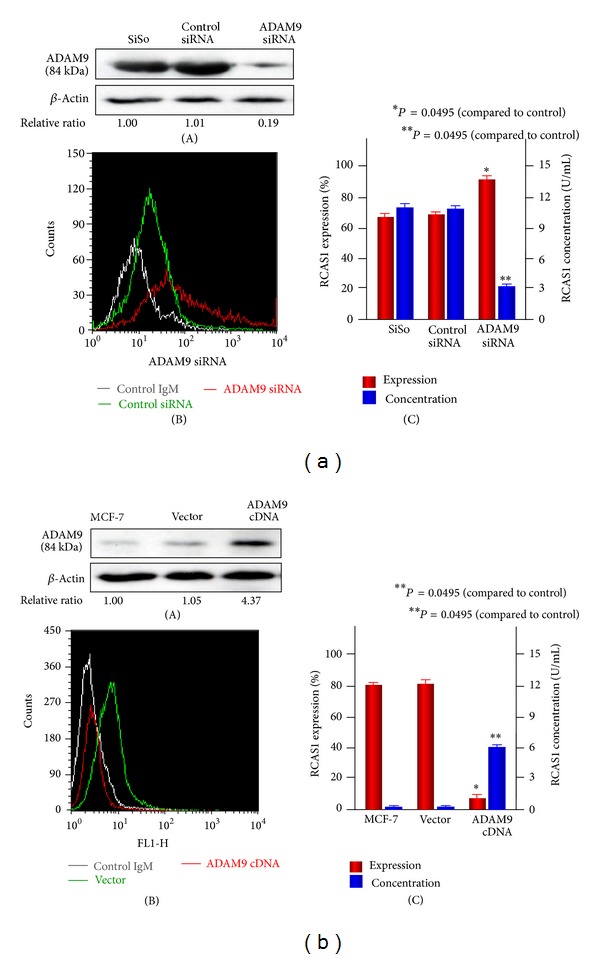
Change in RCAS1 expression and shedding after gene transfection. (a) ADAM9 siRNA transfection in SiSo cells. (A) A Western blot revealed that ADAM9 expression diminished after ADAM9 siRNA transfection. (B) Flow cytometric analysis showed that cell surface expression of RCAS1 increased after ADAM9 siRNA transfection. (C) Transfection of ADAM9 siRNA significantly augmented RCAS1 expression but decreased the RCAS1 concentration in culture supernatants (*P* = 0.0495). (b) ADAM9 cDNA transfection in MCF-7 cells. (A) A Western blot revealed that ADAM9 expression was enhanced after ADAM9 cDNA transfection. (B) Flow cytometric analysis showed that the cell surface expression of RCAS1 decreased after ADAM9 cDNA transfection. (C) Transfection of ADAM9 cDNA significantly diminished RCAS1 expression but increased the concentration of RCAS1 in culture supernatants (*P* = 0.0495). Mean values of triplicate measurements are shown.

ADAM9 expression was knocked down in SiSo cells with siRNA. ADAM9 siRNA-transfected cells showed suppressed ADAM9 expression and inversely increased RCAS1 expression on the cell surface (Figure 1(a) (A) (B)). The RCAS1 expression and concentration were also quantitatively analyzed and shown in Figure 1(a) (C). While transfection of ADAM9 siRNA significantly augmented RCAS1 expression, the amount of RCAS1 in the culture supernatant was markedly decreased (*P* = 0.0495). On the other hand, ADAM9 expression was upregulated in MCF-7 cells following transfection of ADAM9 cDNA (Figure 1(b) (A)). However, RCAS1 expression was significantly reduced, even though RCAS1 shedding was accelerated by induction of ADAM9 expression (Figure 1(b) (B) (C)) (*P* = 0.0495). On the other hand, the ADAM17 expression level was also higher than other proteases. ADAM17 is expressed in various tissues and has been reported to be associated with cancer progression events such as invasion, migration, and metastasis [15]. We also evaluated RCAS1 expression and shedding after ADAM17 gene transfection and found no significant change in either expression or shedding (see Supplementary Figure 1 in Supplementary Material available online at http://dx.doi.org/10.1155/2014/482396). Taken together, these data indicate that ADAM9 rather than ADAM17 is involved in RCAS1 shedding.

### 3.3. Analysis of Apoptotic Cell Death Induced in K562 Cells

**Figure 2:**
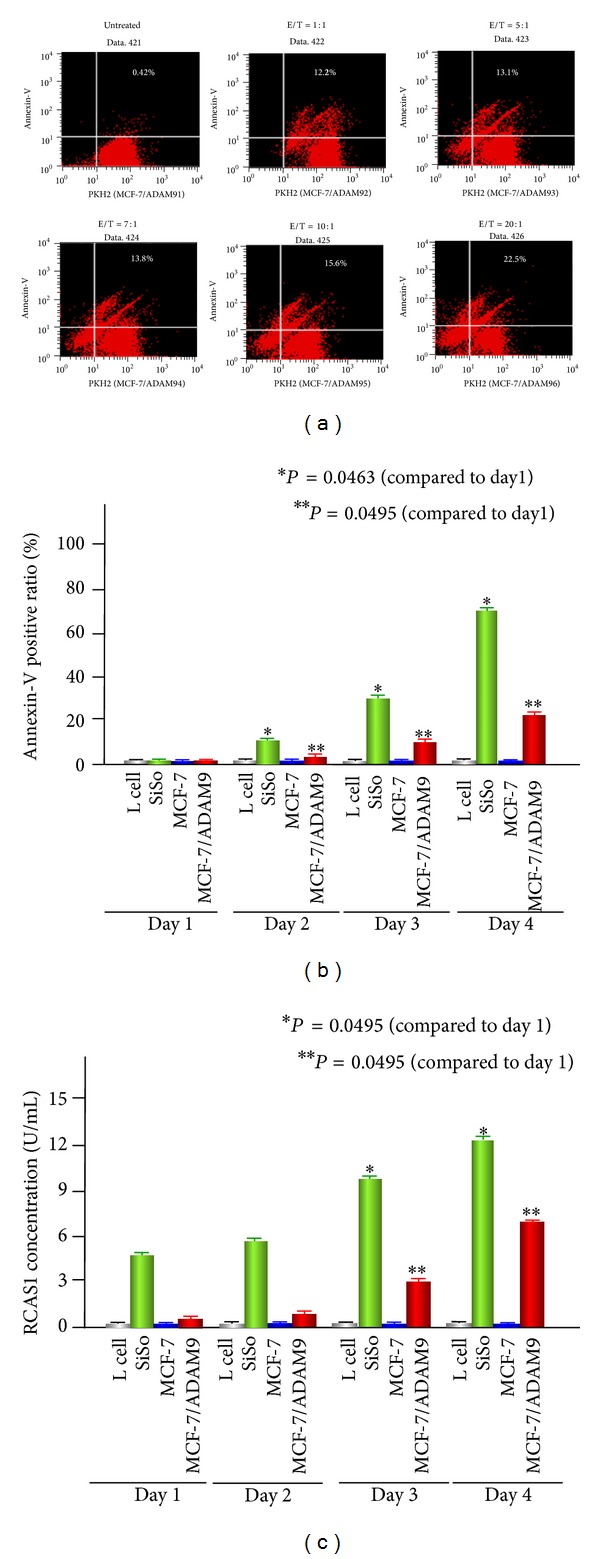
Analysis of apoptotic cell death induced in K562 cells. (a) K562 cell apoptosis was analyzed by flow cytometry. Increases in the annexin-V positive ratio were dependent on the E/T ratio after 4 days of culture. The percentage of cells double positive for PKH2 and annexin-V is indicated. (b) The increase in the number of apoptotic K562 cells was dependent on the culture period (E/T ratio = 20 : 1). Both L cells and MCF-7 cells were used as a negative control and SiSo cells acted as a positive control for inducing apoptosis in K562 cells. (c) The RCAS1 concentration in cell supernatants was measured by ELISA. The RCAS1 level in MCF-7/ADAM9 supernatants increased with the culture time, even though MCF-7 cells alone do not shed RCAS1. Mean values of triplicate measurements are shown.

Apoptosis of K562 cells was induced using a coculture system with SiSo, MCF-7, MCF-7/ADAM9, and L cells. Both L cells and MCF-7 cells were used as a negative control and SiSo cells were a positive control for inducing apoptosis in K562 cells [9]. Although K562 apoptosis was not induced by coculture with MCF-7 cells, the effecter cell MCF-7/ADAM9 could induce apoptosis in K562 cells.  Figure 2(a) shows that the annexin-V positive ratio increased dependently on the E/T ratio after 4 days of culture wherein 22.5% (this number represents the percentage of cells that were double positive for PKH2 and annexin-V) of K562 cells were apoptotic with a 20 : 1 E/T ratio. K562 apoptosis increased significantly depending on the culture period (Figure 2(b)) (*P* = 0.0495).

Next, we measured the RCAS1 concentration in cell supernatants. Although RCAS1 was not detected in L cells and MCF-7 cells, MCF-7/ADAM9 cells shed RCAS1 depending on the length of the culture period (Figure 2(c)). The level of RCAS1 shed from MCF-7/ADAM9 cells was lower than for SiSo cells, but the amount of RCAS1 significantly increased after four days of culture as compared to the first day (*P* = 0.0495). These results suggest that apoptosis of K562 cells was induced by RCAS1 that was shed after ADAM9 proteolysis.

### 3.4. RCAS1 and ADAM9 Expression in Cell Lines and Cancerous Tissues

**Figure 3:**
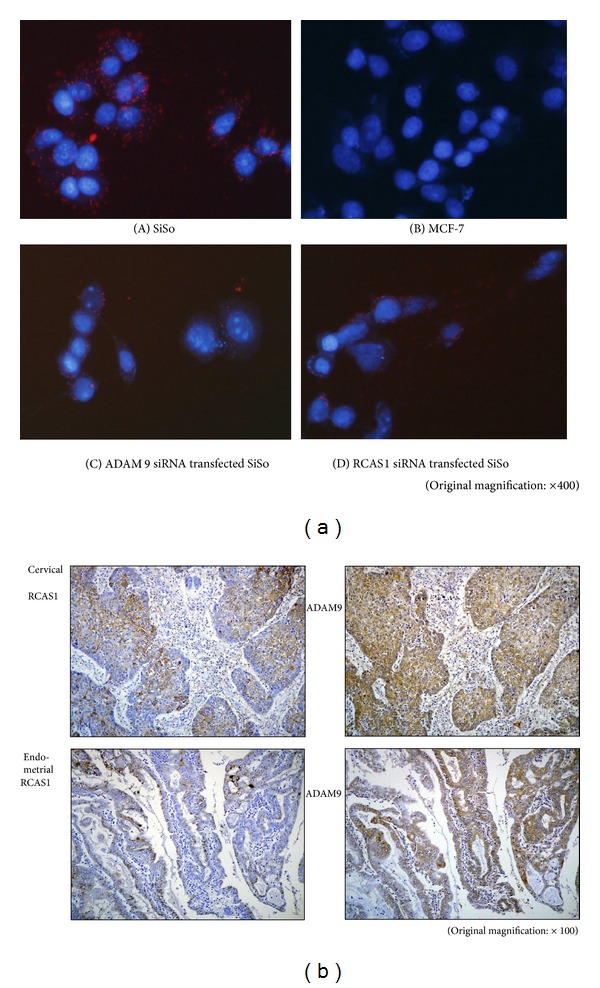
RCAS1 and ADAM9 expression in cell lines and cancerous tissues. (a) RCAS1 and ADAM9 colocalization was immunocytochemically analyzed in SiSo, MCF-7, ADAM9 siRNA-transfected SiSo, and RCAS1 siRNA-transfected SiSo cells using the Duolink detection kit. The red dots indicate RCAS1 and ADAM9 colocalization. (b) The expression pattern of RCAS1 and ADAM9 is shown in one representative case from cervical and endometrial cancer. Diffuse staining for RCAS1 and ADAM9 was observed both in the cytoplasm and on the cell membrane of the cancer cells.

RCAS1 and ADAM9 colocalization was immunocytochemically analyzed in SiSo and MCF-7 cells using a Duolink detection kit. Negative controls without immunized immunoglobulins showed no red dots indicating colocalization of RCAS1 and ADAM9 (Supplementary Figures 2(a) and 2(b)). While red dots were observed in SiSo cells, they were absent in MCF-7 cells (Figure 3(a) (A) (B)). Although red dots in ADAM9 siRNA-transfected SiSo cells and RCAS1 siRNA-transfected SiSo cells rarely occurred (Figure 3(a) (C) (D)), they were occasionally observed in MCF-7/ADAM9 cells (Supplementary Figure 2(e)).

RCAS1 and ADAM9 expression was also evaluated in cancerous tissues by immunohistochemistry. ADAM9 expression was detected in normal cervical epithelium and endometrial glands with weak cytoplasmic staining and membrane immunoreactivity (data not shown). On the other hand, prominent staining for ADAM9 was detected in cervical and endometrial cancer (Figure 3(b)). The difference in ADAM9 protein expression levels between normal epithelium and cancerous tissues was highly significant. Diffuse staining for RCAS1 and ADAM9 was observed both in the cytoplasm and on the cell membrane of cancer cells. Of 47 patients with cervical cancer, 8, 17, 13, and 9 cases showed no expression, 1+, 2+, and 3+ of RCAS1, respectively, while 7, 8, 11, and 21 cases had no expression, 1+, 2+, and 3+, respectively, for ADAM9. Two cases were double-negative and 34 cases were double-positive for RCAS1 and ADAM9. Five cases were single-positive for RCAS1 and 6 cases were single-positive for ADAM9. In 48 patients with endometrial cancer, 16, 14, 12, and 6 cases showed no expression, 1+, 2+, and 3+, respectively, for RCAS1, while for ADAM9, 6, 9, 14, and 19 cases had no expression, 1+, 2+, and 3+, respectively. Two cases were double-negative and 28 cases were double-positive for RCAS1 and ADAM9. Four cases were single-positive for RCAS1 and 14 cases were single-positive for ADAM9. There was no statistically significant association between RCAS1 and ADAM9 expression in both cervical and endometrial cancer.

### 3.5. The Association between Serum RCAS1 Concentration and RCAS1/ADAM9 Expression in Uterine Cancer Patients

**Figure 4:**
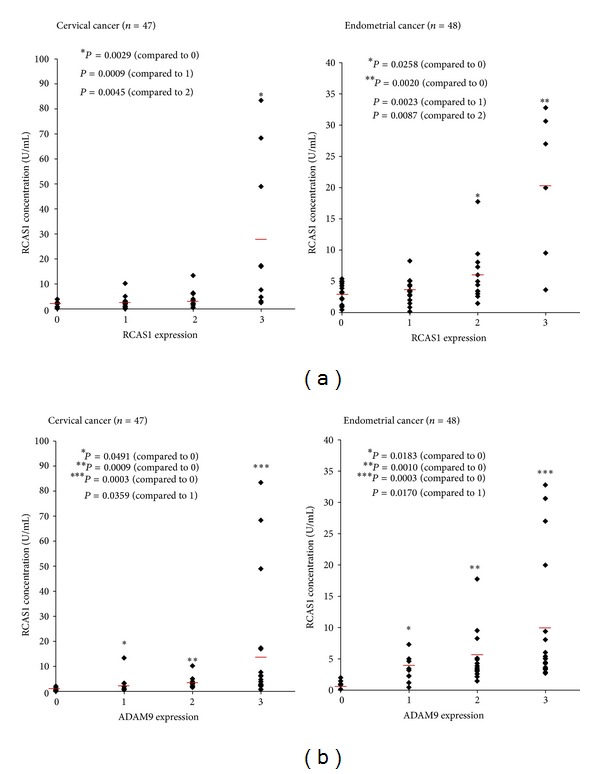
Association between serum RCAS1 concentration and RCAS1/ADAM9 expression in uterine cancer patients. Serum RCAS1 levels significantly increased in a manner that was dependent on both RCAS1 (a) and ADAM9 (b) expression in cervical and endometrial cancer patients. Mean values of triplicate measurements are shown.

We evaluated the association between serum RCAS1 concentration and RCAS1/ADAM9 expression in 47 cervical and 48 endometrial cancer patients. Serum RCAS1 levels significantly increased in a manner that was dependent on RCAS1 and ADAM9 expression in both cancer types (Figures 4(a) and 4(b)). These data further support a role for ADAM9 in regulating RCAS1 shedding in human uterine cancer.

## 4. Discussion

This is the first report showing that ADAM9 is involved in RCAS1 ectodomain shedding. ADAM9 is a member of the ADAM protein family, for which 40 gene members have currently been identified with 21 members being functional in humans [23]. ADAMs are membrane-anchored glycoproteins that consist of pro- and metalloprotease, disintegrin, cysteine-rich, EGF-like, and cytoplasmic domains, which enable these proteins to have a versatile range of physiological and pathological functions [24]. Some ADAMs participate in fertilization, myogenesis, neurogenesis, and activation of growth factors/immune regulators such as tumor necrosis factor (TNF)-*α* [25]. On the other hand, specific ADAMs have been implicated in a number of diseases, including rheumatoid arthritis, Alzheimer's disease, atherosclerosis, asthma, and cancer [17, 26]. ADAM9 was cloned and sequenced by Weskamp et al. in 1996 [27]. ADAM9 is widely expressed in the human body and is a catalytically active metalloprotease-disintegrin protein that has been implicated in the ectodomain cleavage of heparin-binding (HB)-EGF and as an *α*-secretase for the amyloid precursor protein [17]. Olson et al. demonstrated the reproductive stage-specific expression of ADAM9 mRNA in rabbit uterine epithelium during the peri-implantation period [28]. ADAM9 expression is upregulated as progesterone levels rise and at blastocyst implantation sites. ADAM9 also plays a pivotal role in some signaling pathways, wherein transmission of information might induce some inconvertible exacerbations of disease [29]. ADAM9 is reportedly involved in several human diseases such as inflammatory disorders, oxygen-induced retinopathy (OIR), and cancer [30]. ADAM9 expression was found to be upregulated in various solid tumors and is often associated with adverse prognostic parameters or shorter patient survival times. ADAM9 overexpression was reported in several human carcinomas, including oral [31], lung [32], breast [21, 33], stomach [34], liver [35], pancreas [36], colon [37], kidney [38], prostate [39], cervix [40], and melanoma [41], and is correlated with cancer progression and metastasis, as well as having a predictive capacity for patient survival times. The background for the clinical significance of ADAM9 in tumor progression has been investigated by* in vitro* experiments. ADAM9 expression was found to be elevated in a cell line having high metastatic potential as compared to cell lines that had a low metastatic potential [35]. Enhanced ADAM9 expression induced by gene transfection also promoted cell invasion [37]. ADAM9 is a secreted protein and its soluble form promoted the invasive phenotype of carcinoma cell lines by binding to the *α*6*β*4 and *α*2*β*1 integrins on the surface of carcinoma cells through its disintegrin domain [42]. In melanoma and a hepatic metastatic site of colon cancer, ADAM9 expression was upregulated at the invasion front, again supporting its role in tumor progression [41, 42].

Zubel et al. previously reported that ADAM9 is expressed in cervical squamous cell carcinoma [40]. In this study, the positive ratio of ADAM9 was 87% (29 out of 33 cases) and 78% (11 out of 14 cases) in squamous cell- and adenocarcinoma, respectively, which are values that are somewhat lower than the 93% (13 out of 14 cases) given in the previous report. On the other hand, this is the first report concerning ADAM9 expression in endometrial cancer wherein ADAM9 positivity was seen in 87% of cases (42 out of 48 cases) and ADAM9 expression was strong (3+) in 19 of 48 cases.

Although cancer patients currently receive multidisciplinary therapies that integrate surgery, radiation, and chemotherapy, limitations in the efficacy of anticancer treatments against advanced or recurrent tumors require the development of novel and highly specific targets for therapy. Considering the significance of tumor progression, RCAS1 has potential value as a unique biomarker and molecular target for diagnostics and therapy. Several therapeutic strategies should thus be considered to suppress the expression and function of RCAS1. A first strategy could be to modulate RCAS1 expression using siRNA. The value of this technique was shown in studies where the induction of molecular-specific siRNA into tumor cells reduced T lymphocyte apoptosis and VEGF secretion, which was followed by tumor regression [8, 43]. A second strategy for inhibiting RCAS1 function is to use antibodies. Serum from uterine and ovarian cancer patients inhibited growth of RCAS1 putative receptor expressing K562 cells, and this suppressive effect could be partially negated after immunoprecipitation to remove RCAS1 [19, 44]. A third strategy would be to modulate ADAM9-mediated RCAS1 ectodomain shedding. Soluble RCAS1, rather than the membrane-anchored form, is mainly responsible for inducing apoptosis [9]. In this study, we did not use a catalytic inactive mutant form of ADAM9 to demonstrate that the proteolytic activity of ADAM9 is directly responsible for RCAS1 shedding. Thus it is possible that ADAM9 indirectly controls RCAS1 processing. To date, several methods have been reported to suppress ADAM9 expression and function. Knockdown of ADAM9 by RNA interference resulted in reduced cell proliferation, invasion, and metastasis [35, 39, 45], as well as increased sensitivity to radiation and chemotherapeutic drugs [46]. Blocking of ADAM9 activity with specific antibodies resulted in inhibited cell growth of gastric cancer cell lines [34]. Reactive oxygen species (ROS) can also induce expression of ADAM9 via p38 mitogen-activated protein kinase activation [47]. Sung et al. observed apoptotic cell death in prostate cancer cells by decreasing ADAM9 expression via the administration of an antioxidant or genetic transfer of a hydrogen peroxide degradative enzyme [39]. Moreover, Moss et al. described that the metalloproteinase inhibitor marimastat is potent against ADAM9 [48].

Before initiating molecular targeting therapy, selection of eligible patients is necessary. For cervical and endometrial cancer in which RCAS1 is a clinical prognostic factor [2], tissue sampling and expression analysis of RCAS1 and ADAM9 can be easily performed. While several selective synthetic inhibitors that are active against a small number of ADAMs have recently been described [49, 50], adverse effects induced by targeting therapy can be a significant concern when the targeted molecules are ubiquitously expressed. Weskamp et al. generated mice lacking ADAM9 to learn more about the function of this protein during development and in adults [51]. During mouse development, ADAM9 mRNA is ubiquitously expressed, with particularly high expression levels in the developing mesenchyme, heart and brain. Despite the ubiquitous expression of ADAM9, these knockout mice appear to develop normally, are viable and fertile, and have no major pathological phenotypes compared to wild-type mice. Therefore, potential adverse effects produced by targeting ADAM9 activity could be anticipated to be tolerable.

Some recent advances might offer in the near future the opportunity to design such specific inhibitors using, for example, siRNAs or monoclonal antibodies. The precise understanding of the exact role played by RCAS1 and ADAM9 in cancer appears to be of particular importance from the perspective of designing new therapeutic strategies that are based on the control or inhibition of these proteins.

## 5. Conclusion

In several human malignancies, RCAS1 expression is associated with aggressive characteristics and poor overall survival. Since RCAS1 promotes tumor cell evasion of immune surveillance by inducing apoptosis in immune cells and also remodels the cancer stromal microenvironment, RCAS1 is believed to contribute to tumor progression. Soluble RCAS1, rather than the membrane-anchored form, is mainly responsible for inducing immune cell apoptosis. ADAM9 is involved in RCAS1 ectodomain shedding; therefore, inhibition of ADAM9 activity might contribute to controlling the biological functions of RCAS1. A precise understanding of the role played by RCAS1 and ADAM9 is essential to design novel strategies to treat cancer.

## Supplementary Material

Supplementary figure 1: Change in RCAS1 expression and shedding after ADAM17 gene transfection.
Supplementary figure 2: RCAS1 and ADAM9 expression in cell lines.

## Figures and Tables

**Table 1:** Clinicopathologic variables for uterine cancer patients.

Clinicopathologic variables	Number of patients
*Cervical cancer *	
Age (years; mean ± SD)	43 ± 11
Stage	
I	36
II	11
Histologic subtype	
Squamous cell carcinoma	33
Adenocarcinoma	14
*Endometrial cancer *	
Age (years; mean ± SD)	58 ± 11
Stage	
I	28
II	5
III	13
IV	2
Grade	
1	24
2	15
3	9

**Table 2:** Microarray data on proteases.

Gene symbol	Probe ID	Probe set ID	SiSo signal	MCF-7 signal	SiSo/MCF-7
ADAM2	HU133p2_17106	207664_at	7.0	6.3	1.11
ADAM3A	HU133p2_26379	217090_at	1.4	1.9	0.74
ADAM5	HU133p2_26289	216998_s_at	2.9	7.2	0.40
ADAM6	HU133p2_47159	237909_at	3.1	7.1	0.44
ADAM7	HU133p2_20597	211239_s_at	7.3	3.0	2.43
ADAM8	HU133p2_14627	205179_s_at	12.3	8.4	1.46
ADAM9	HU133p2_11830	202381_at	1856.6	275.1	6.75
ADAM10	HU133p2_06424	1562137_at	5.2	4.7	1.11
ADAM11	HU133p2_49087	239837_at	9.6	8.2	1.17
ADAM12	HU133p2_51710	242460_at	9.2	6.4	1.44
ADAM15	HU133p2_26298	217007_s_at	83.6	43.4	1.93
ADAM17	HU133p2_22834	213532_at	117.7	195.5	0.60
ADAM18	HU133p2_17039	207597_at	5.6	8.3	0.67
ADAM19	HU133p2_30412	221128_at	8.9	13.3	0.67
ADAM20	HU133p2_16866	207423_s_at	7.7	18.7	0.41
ADAM21	HU133p2_17107	207665_at	6.8	6.2	1.10
ADAM22	HU133p2_53445	244194_at	5.9	5.6	1.05
ADAM23	HU133p2_15493	206046_at	6.2	24.0	0.26
ADAM28	HU133p2_17694	208269_s_at	1.6	7.4	0.22
ADAM29	HU133p2_30621	221337_s_at	0.4	9.1	0.04
ADAM30	HU133p2_30730	221446_at	3.7	9.5	0.39
ADAM32	HU133p2_00020	1552266_at	11.3	10.4	1.09
ADAM33	HU133p2_43119	233868_x_at	33.1	31.9	1.04
ADAMTS1	HU133p2_31443	222162_s_at	0.6	4.4	0.14
ADAMTS2	HU133p2_23835	214535_s_at	3.6	2.3	1.57
ADAMTS3	HU133p2_24209	214913_at	4.5	5.3	0.85
ADAMTS4	HU133p2_02285	1555380_at	13.9	16.1	0.86
ADAMTS5	HU133p2_29220	219935_at	2.2	2.4	0.92
ADAMTS6	HU133p2_09800	1570351_at	3.5	8.2	0.43
ADAMTS7	HU133p2_29991	220706_at	2.2	5.9	0.37
ADAMTS8	HU133p2_29962	220677_s_at	8.4	12.4	0.68
ADAMTS9	HU133p2_03368	1556989_at	6.4	11.1	0.58
ADAMTS10	HU133p2_41388	232133_at	1.2	0.8	1.50
ADAMTS12	HU133p2_30705	221421_s_at	13.3	19.8	0.67
ADAMTS13	HU133p2_33121	223844_at	3.0	10.1	0.30
ADAMTS15	HU133p2_00845	1553427_at	2.0	1.5	1.33
ADAMTS16 ADAMTS17	HU133p2_46339 HU133p2_00332	237089_at 1552725_s_at	2.2 9.9	2.4 17.0	0.92 0.58
ADAMTS18	HU133p2_00702	1553234_at	9.1	10.7	0.85
ADAMTS19	HU133p2_00663	1553180_at	4.8	26.0	0.18
ADAMTS20 ADAMDEC1	HU133p2_30002 HU133p2_15581	220717_at 206134_at	2.4 0.5	4.6 4.5	0.52 0.11
ADAMTSL1	HU133p2_33638	224371_at	1.0	2.1	0.48
ADAMTSL2	HU133p2_16076	206629_at	1.2	1.2	1.00
ADAMTSL3	HU133p2_04979	1559748_at	2.2	12.0	0.18
MMP1	HU133p2_13923	204475_at	35.0	46.0	0.76
MMP2	HU133p2_08387	1566677_at	1.2	0.4	3.00
MMP3	HU133p2_15276	205828_at	19.3	25.0	0.77
MMP7	HU133p2_13707	204259_at	17.9	5.4	3.31
MMP8	HU133p2_16774	207329_at	0.6	0.4	1.50
MMP9	HU133p2_13384	203936_s_at	21.2	43.1	0.49
MMP10	HU133p2_15128	205680_at	1.1	10.1	0.11
MMP11	HU133p2_45158	235908_at	2.3	14.4	0.16
MMP12	HU133p2_14028	204580_at	3.0	3.9	0.77
MMP13	HU133p2_15407	205959_at	7.9	10.5	0.75
MMP14	HU133p2_09936	160020_at	18.6	28.6	0.65
MMP15	HU133p2_53134	243883_at	1.2	1.6	0.75
MMP16	HU133p2_17594	208166_at	3.7	4.7	0.79
MMP17	HU133p2_15681	206234_s_at	6.9	28.0	0.25
MMP19	HU133p2_14022	204574_s_at	2.3	4.7	0.49
MMP20	HU133p2_17041	207599_at	1.9	0.9	2.11
MMP21	HU133p2_00237	1552592_at	3.8	0.8	4.75
MMP23A/B	HU133p2_16565	207118_s_at	1.4	0.6	2.33
MMP24	HU133p2_17809	208387_s_at	5.0	5.7	0.88
MMP25	HU133p2_49304	240054_at	14.4	34.8	0.41
MMP26	HU133p2_29826	220541_at	4.1	7.7	0.53
MMP27	HU133p2_30068	220783_at	1.1	0.7	1.57
MMP28	HU133p2_33479	224207_x_at	14.8	23.3	0.64
MMPL1	HU133p2_16735	207289_at	8.7	9.6	0.91
