# Supplementary material for: A Disintegrin and Metalloproteinase 9 Is Involved in Ectodomain Shedding of Receptor-Binding Cancer Antigen Expressed on SiSo Cells
Source: Biomed Res Int. 2014 Aug 7;2014:482396. doi: 10.1155/2014/482396 (PMC4142186; doi:10.1155/2014/482396)

**Supplementary figure legends**

Supplementary figure 1.

Change in RCAS1 expression and shedding after ADAM17 gene transfection. To construct specific siRNA for ADAM17, oligonucleotides were synthesized and purified by Takara Bio (Shiga, Japan) as follows: sense 5’-CCACUUUGGAGAUUUGUUATT-3’, and antisense 5’-UAACAAAUCUCCAAAGUGGTT-3’. The control siRNA GC content was 33.3%, which is identical to that of the siRNAs constructed here. MCF-7 cells were transfected with the expression vector pCMV6 that contained human ADAM17 cDNA or vector alone (OriGene Technologies, Rockville, MD). For Western blot, chicken anti-ADAM17 (R&D Systems, Minneapolis, MN) antibody and peroxidase-conjugated goat anti-chicken IgG (Novus Biologicals) was used as primary and secondary antibody, respectively. (A) ADAM17 siRNA transfection in SiSo cells. (a) A Western blot revealed that ADAM17 expression diminished after ADAM17 siRNA transfection. (b) Flow cytometric analysis showed that cell surface expression of RCAS1 did not change after ADAM17 siRNA transfection. (c) Transfection of ADAM17 siRNA did not alter either expression or shedding of RCAS1. (B) ADAM17 cDNA transfection in MCF-7 cells. (a) A Western blot revealed that ADAM17 expression was enhanced after ADAM17 cDNA transfection. (b) Flow cytometric analysis showed that the cell surface expression of RCAS1 did not change after ADAM17 cDNA transfection. (c) Transfection of ADAM17 cDNA did not alter either expression or shedding of RCAS1. Mean values of triplicate measurements are shown.

Supplementary figure 2.

RCAS1 and ADAM9 expression in cell lines. RCAS1 and ADAM9 co-localization was immunocytochemically analyzed using the Duolink detection kit. The red dots indicate RCAS1 and ADAM9 co-localization. (A) SiSo cells were stained without immunized immunoglobulins as a negative control. No red dots were seen. (B) MCF-7 cells were stained without immunized immunoglobulins as a negative control. No red dots were seen. (C) MCF-7/ADAM9 cells occasionally revealed red dots.


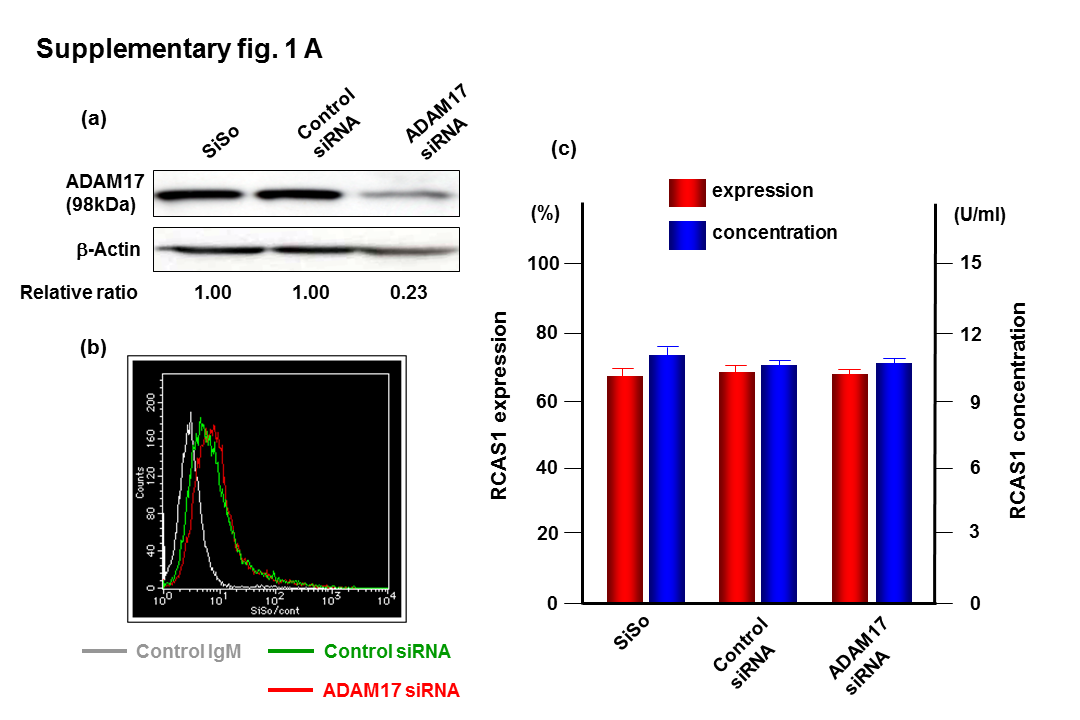


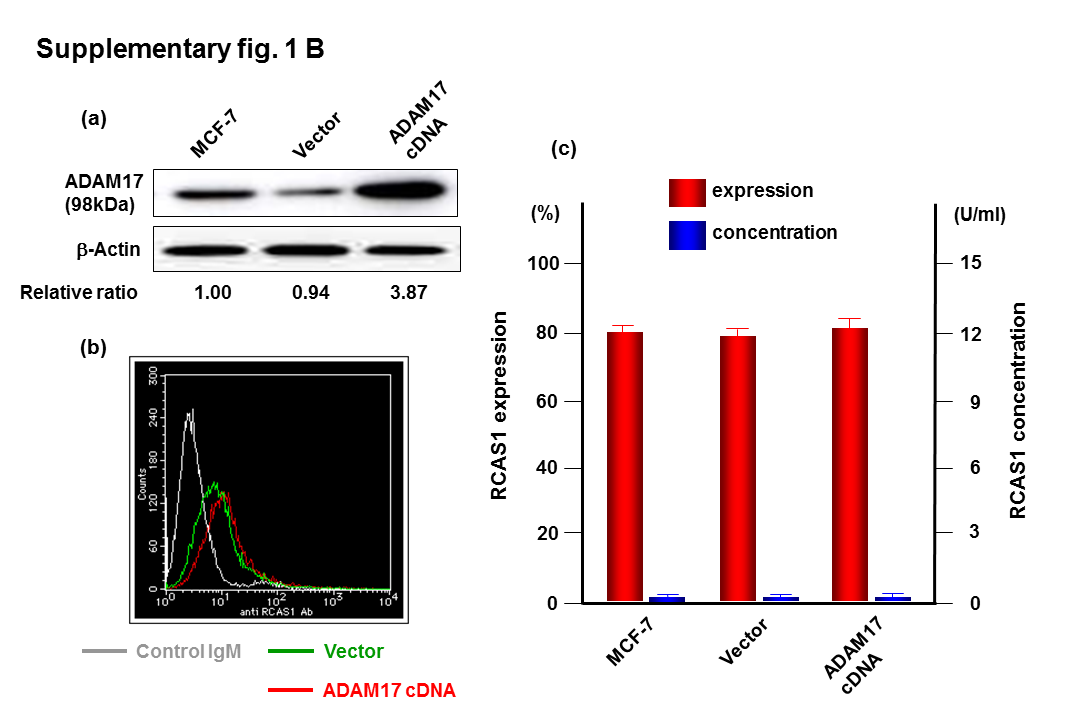


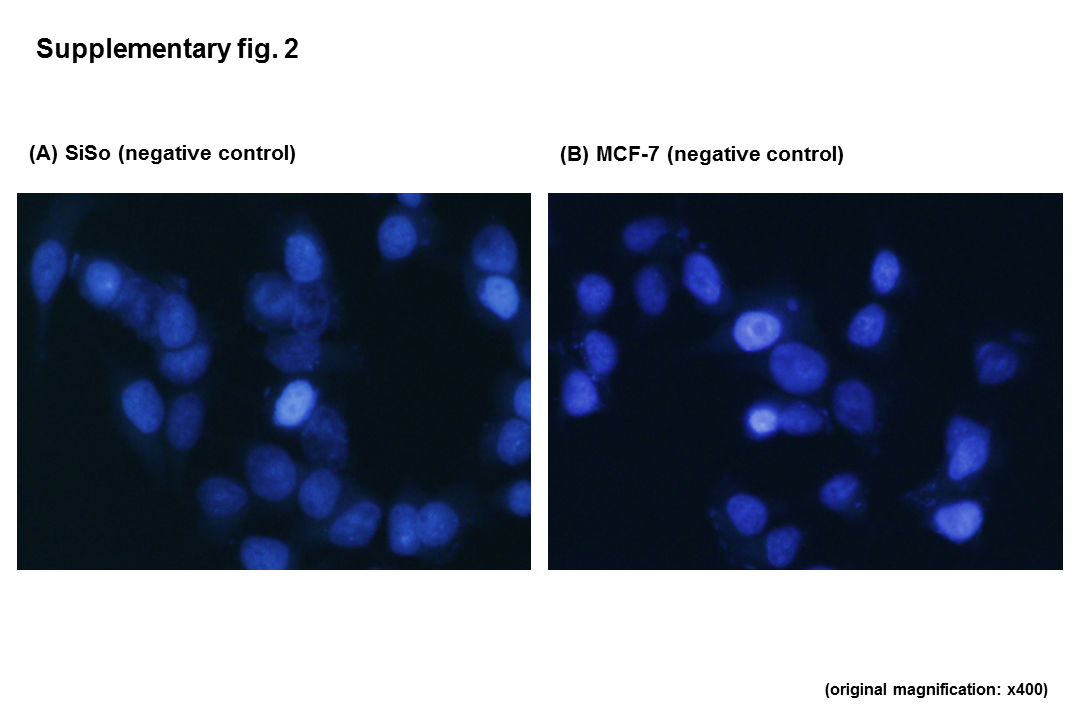


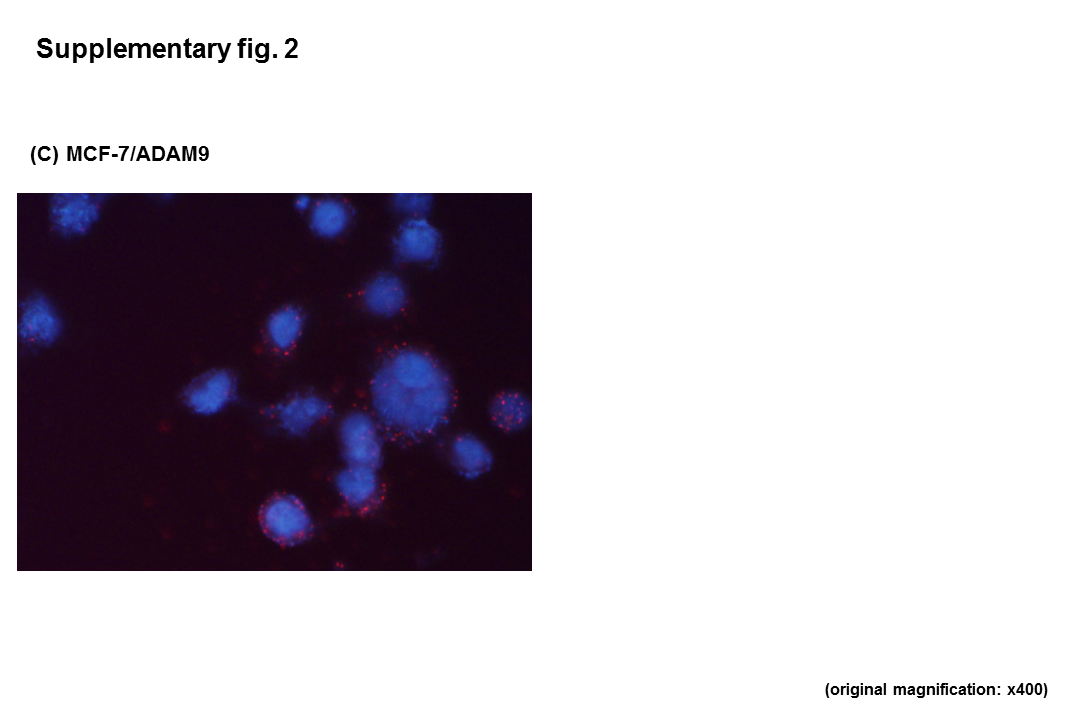

Supplement: Supplementary file 1 — Supplementary figure 1: Change in RCAS1 expression and shedding after ADAM17 gene transfection. Supplementary figure 2: RCAS1 and ADAM9 expression in cell lines. [file 482396.f1.docx]
